# Factors of Severity in Patients with COVID-19: Cytokine/Chemokine Concentrations, Viral Load, and Antibody Responses

**DOI:** 10.4269/ajtmh.20-1110

**Published:** 2020-10-27

**Authors:** Ji-Soo Kwon, Ji Yeun Kim, Min-Chul Kim, Se Yoon Park, Baek-Nam Kim, Seongman Bae, Hye Hee Cha, Jiwon Jung, Min-Jae Kim, Myung Jin Lee, Seong-Ho Choi, Jin-Won Chung, Eui-Cheol Shin, Sung-Han Kim

**Affiliations:** 1 Graduate School of Medical Science and Engineering, Korea Advanced Institute of Science and Technology, Daejeon, Republic of Korea;; 2 Department of Infectious Diseases, Asan Medical Center, University of Ulsan College of Medicine, Seoul, Republic of Korea;; 3 Division of Infectious Diseases, Department of Internal Medicine, Chung-Ang University Hospital, Chung-Ang University College of Medicine, Seoul, Republic of Korea;; 4 Department of Infectious Diseases, Soonchunhyang University Seoul Hospital, Soonchunhyang University College of Medicine, Seoul, Republic of Korea;; 5 Department of Infectious Diseases, Inje University Sanggye Paik Hospital, Inje University College of Medicine, Seoul, Republic of Korea

## Abstract

The severity of COVID-19 ranges from mild to critical diseases. However, limited data have been published on the detailed kinetics of viral load and host immune response throughout the disease course depending on disease severity. In this study, we comprehensively analyzed viral load, antibody responses to SARS-CoV-2, and cytokines/chemokines during the disease course, and identified the factors related to severity. Nasopharyngeal (NP) and plasma specimens were obtained from 31 patients with COVID-19 during hospitalization. Viral RNA in NP specimens was quantified by reverse transcription–PCR. Anti–SARS-CoV-2 antibodies and cytokines/chemokines in plasma specimens were analyzed by ELISA and cytometric bead array. The viral load in patients with COVID-19 peaked at the early stage of the disease and continuously decreased. Severe and critical cases showed higher viral load and prolonged viral shedding than asymptomatic and mild cases. Whereas plasma IgG was gradually increased and maintained during hospitalization, plasma IgM peaked at 3 weeks after symptom onset and dissipated. The antibody response in severe and critical cases was slightly delayed but stronger than those in others. High levels of interferon (IFN)-α, IFN-γ–induced protein-10, monokine induced by IFN-γ, and interleukin-6 at 5–10 days from symptom onset were associated with the severity of COVID-19. Our data indicate that high viral load in the respiratory tract and excessive production of cytokines and chemokines between 1 and 2 weeks from the symptom onset were significantly associated with the severity of COVID-19.

## INTRODUCTION

In December 2019, cases of pneumonia with unknown etiology were reported in Wuhan, China. In January 2020, a novel coronavirus was identified as the cause of the pneumonia, and its genome sequence had 79% identity to SARS-CoV; as such, the virus was designated as SARS-CoV-2 and the disease it causes as COVID-19.^1,2^ The WHO declared the outbreak of COVID-19 as a worldwide pandemic in March 2020.

Previous studies on the epidemiology and clinical features of COVID-19 have shown that SARS-CoV-2 infection usually results in mild disease, although some patients occasionally develop severe or critical illness.^3^ Poor clinical outcomes of COVID-19 has been associated with older age, male gender, and the presence of underlying conditions such as hypertension, obesity, and type 2 diabetes mellitus.^4–6^ However, the pathophysiologic mechanisms by which SARS-CoV-2 causes severe illness are largely unknown. Our previous single-cell RNA sequencing study revealed that monocytes from patients with severe COVID-19 exhibit increased type I interferon (IFN) response in addition to tumor necrosis factor (TNF)/interleukin (IL)-1β–driven inflammation^7^; by contrast, others reported that severe COVID-19 was associated with impaired type I IFN response with an excessive release of TNF-α and IL-6.^8^

However, previous studies do not reflect the cytokine responses during the various courses of the disease. In this study, we comprehensively analyzed the viral load, antibody responses to SARS-CoV-2, and cytokine/chemokine profiles during the disease course and identified the factors related to the severity of COVID-19.

## MATERIALS AND METHODS

### Patients and clinical samples.

We prospectively enrolled 31 confirmed cases of COVID-19 admitted to four university-affiliated hospitals in the Republic of Korea—Asan Medical Center, Chung-Ang University Hospital, Soonchunhyang University Seoul Hospital, and Inje University Sanggye Paik Hospital—from February 2020 to April 2020. COVID-19 was confirmed by real-time reverse transcription–PCR (RT-PCR) analysis for the RdRp gene of SARS-CoV-2. The severity of COVID-19 was categorized into mild, moderate, severe, and critical according to the WHO guidance.^9^ Peripheral blood was collected at admission and every 2–3 days thereafter until discharge. Plasma samples were immediately separated by centrifugation and stored at −70°C until further analysis; for cytokine and antibody analysis, approximately 1 mL of plasma was irradiated with up to 6 million rad from a^60^Co gamma source according to the method used in our previous study.^10^ The study was approved by the respective institutional review boards of each participating hospital.

### SARS-CoV-2 RT-PCR.

Viral RNA was extracted from the upper respiratory tract swab samples using the MagNA Pure 96 system (Roche Diagnostics, Mannheim, Germany) according to the manufacturer’s instructions. RNA was analyzed with the PowerChek 2019-nCoV Real-Time PCR Kit (KogeneBiotech, Seoul, Republic of Korea) that targets the RdRp gene of SARS-CoV-2 and the E gene of beta-coronavirus, and the Allplex^™^ 2019-nCoV assay (Seegene) that targets the RdRp gene and N gene of SARS-CoV-2 and the E gene of beta-coronavirus. Ct values < 40 for RdRp gene were considered as positive results.

### SARS-CoV-2 serology.

We measured the levels of human anti–SARS-CoV-2 IgG and IgM by using laboratory-developed ELISA. SARS-CoV-2 S1-His protein (Sino Biological, Beijing, China) was coated onto 96-well plates (MaxiSorp, Thermo Fisher Scientific, Waltham, MA) at a concentration of 2 μg/mL in PBS. Plasma samples were used at dilutions of 1:100, 1:1,000, and 1:10,000. horseradish peroxidase-conjugated antihuman IgG (Jackson Immunoresearch, West Grove, PA) and IgM (MilliporeSigma, Burlington, MA) were used as secondary antibodies. The optical density (OD) value of 450 nm (OD_450_) was measured. If OD_450_ of 1/100 or 1/1,000 diluted sample exceeds 2.5, the OD_450_ of next diluted sample was multiplied by dilution factor (OD_450_ ratio) and determined as the endpoint titer. The OD_450_ of plasma specimens that were not previously exposed to SARS-CoV-2 were used to determine the cutoff value (i.e., OD_450_ = 0.4) for both IgG and IgM.

### Cytokine analysis.

Collected plasma samples were stored at −70°C until analysis and analyzed at once, on the same day. We simultaneously measured plasma concentrations of granulocyte colony-stimulating factor, granulocyte-macrophage colony-stimulating factor, IFN-α, IFN-γ, TNF-α, IL-1β, IL-2, IL-6, IL-7, IL-8, IL-10, IL-12p70, IL-13, IL-17A, monocyte chemotactic protein (MCP)-1, macrophage inflammatory protein (MIP)-1α, MIP-1β, regulated on activation and normally T-cell expressed and secreted, monokine induced by IFN-γ (MIG), IFN-γ–induced protein (IP)-10, and vascular endothelial growth factor (VEGF) using a cytometric bead array based on microspheres for detecting cytokine/chemokine according to the manufacturer’s instruction (BD Bioscience, San Jose, CA). Data were acquired using the FACS CANTO II flow cytometer, FACSDiva software (BD Bioscience), and FlowJo software (FlowJo LLC, Ashland, OR), as described in our previous study.^10,11^

### Statistical analyses.

Categorical variables were compared using the Fisher’s exact test or the χ^2^ test, and continuous variables were compared with the Kruskal–Wallis test, Mann–Whitney *U* test, or unpaired Student’s *t*-test. All tests of significance were two-tailed and *P*-values less than 0.05 were considered statistically significant. Statistical analyses were performed using GraphPad Prism 8.4.3 (GraphPad Software, Inc., LA Jolla, CA).

## RESULTS

### Clinical characteristics and outcomes.

A total of 31 patients with COVID-19 confirmed by SARS-CoV-2–specific RT-PCR of Nasopharyngeal (NP) swab specimen were enrolled in this study. The patients were classified according to disease severity: five (16%) mild, 17 (55%) moderate, six (19%) severe, and two (7%) critical patients; one (3%) patient did not have any signs or symptoms and was therefore classified as an asymptomatic case (Table 1); for analysis, the patients were categorized into three groups: asymptomatic and mild (19%), moderate (55%), and severe and critical (26%). Of the 31 patients, 18 (58%) were female, and the mean age (±SD) was 50.0 (±3.3) years. The median time interquartile range (IQR) from symptom onset to admission was 6 days (3–8). The median time (IQR) from admission to discharge was 24 (18–33) days. Old age, initial low WBC count, low platelet count, high CRP level, and fever were identified as factors associated with severity. Detailed baseline characteristics, laboratory tests, and outcomes are shown in Tables 1 and 2.

**Table 1:** Baseline characteristics and outcomes in 31 patients with COVID-19

Variable	Total (*n* = 31), %	Asymptomatic and mild (*n* = 6)	Moderate (*n* = 17), %	Severe and critical (*n* = 8)	*P*-value (asymptomatic and mild vs. moderate)	*P*-value (asymptomatic and mild vs severe and critical)	*P*-value (moderate vs. severe and critical)
Age, mean (±SD) (years)	50.0 (±3.3)	32.8 (±9.5)	45.8 (±17.0)	71.9 (±12.9)	0.105	< 0.001	< 0.001
Female gender	18/31 (58)	3/6	11/17 (65)	4/8	0.521	0.236	0.665
Underlying condition or illness							
Diabetes mellitus	4/31 (13)	0/6	1/17 (6)	3/8	1.000	0.542	0.081
Hypertension	9/31 (29)	1/6	2/17 (12)	6/8	1.000	0.103	0.004
Chronic lung disease	2/31 (6)	0/6	0/17	2/8	1.000	0.473	0.093
Chronic liver disease	1/31 (3)	0/6	1/17 (6)	0/8	1.000	1.000	1.000
Obesity (body mass index > 25)	1/31 (3)	0/6	0/17	1/8	1.000	1.000	0.320
Smoking	2/31 (6)	2/6	0/17	0/8	0.059	0.165	1.000
Symptoms and signs at admission							
Fever	17/31 (55)	0/6	9/17 (53)	8/8	0.048	< 0.001	0.026
Chill	2/19 (11)	0/3*	2/13† (15)	0/3‡	1.000	1.000	1.000
Cough	20/31 (65)	2/6	10/17 (59)	8/8	0.371	0.007	0.057
Sputum	11/31 (35)	1/6	5/17 (29)	5/8	1.000	0.138	1.000
Sore throat	7/31 (23)	0/6	4/17 (24)	3/8	0.539	0.209	0.194
Dyspnea	2/31 (6)	0/6	0/17	2/8	1.000	0.473	0.093
Rhinorrhea	2/31 (6)	1/6	1/17 (6)	0/8	0.463	0.429	1.000
Chest pain	2/31 (6)	2/6	0/17	0/8	0.059	0.165	1.000
Headache	5/31 (16)	0/6	3/17 (18)	2/8	0.539	0.473	1.000
Myalgia	7/31 (23)	0/6	3/17 (18)	4/8	0.539	0.849	0.156
Nasal congestion	2/31 (6)	1/6	1/17 (6)	0/8	0.463	0.429	1.000
Hyposmia	6/31 (19)	1/6	5/17 (29)	0/8	1.000	0.429	0.140
Hypogeusia	5/31 (16)	1/6	4/17 (24)	0/8	1.000	0.429	0.239
Pneumonia	23/31 (74)	0/6	15/17 (88)	8/8	< 0.001	< 0.001	1.000
Time from symptom onset to admission	6 (3–8)	2 (0–4)	6 (3–8)	8 (8–9)	0.005	< 0.001	0.024
Hospital stay (interquartile range) (days)	24 (18–33)	19 (10–28)	27 (21–33)	24 (19–29)	0.182	0.361	0.739
Treatment							
Lopinavir/ritonavir	15/31 (48)	0/6	8/17 (47)	7/8	0.058	0.005	0.088
Hydroxychloroquine	14/31 (45)	4/6	5/17 (29)	5/8	0.162	1.000	0.194
Steroid	1/31 (3)	0/6	0/17	1/8	1.000	1.000	0.320
Antibiotics	6/31 (19)	0/6	0/17	6/8	1.000	0.001	< 0.001
Plasmapheresis	1/31 (3)	0/6	0/17	1/8	1.000	1.000	0.320
Pneumonia during hospitalization	25/31 (81)	0/6	17/17 (100)	8/8	< 0.001	< 0.001	1.000
O_2_ supply	8/31 (26)	0/6	0/17	8/8	1.000	< 0.001	< 0.001
Respiratory failure	2/31 (6)	0/6	0/17	2/8	1.000	0.473	0.093
Septic shock	2/31 (6)	0/6	0/17	2/8	1.000	0.473	0.093
Multiple organ failure	1/31 (3)	0/6	0/17	1/8	1.000	1.000	0.320
Intensive care unit care	3/31 (10)	0/6	0/17	3/8	1.000	0.209	0.024
Mechanical ventilation	2/31 (6)	0/6	0/17	2/8	1.000	0.473	0.093
Extracorporeal membrane oxygenation	1/31 (3)	0/6	0/17	1/8	1.000	1.000	0.320

Values in parentheses indicate percentage of patients positive for variables in each group.

* Not checked in three asymptomatic and mild.

† Not checked in four moderate.

‡ Not checked in five severe and critical.

**Table 2:** Laboratory tests, cytokine/chemokine, viral load, antibody responses, and clinical outcomes in patients with COVID-19

Variable	Asymptomatic and mild (*n* = 6)	Moderate (*n* = 17)	Severe and critical (*n* = 8)	*P*-value (asymptomatic and mild vs. moderate)	*P*-value (asymptomatic and mild vs. severe and critical)	*P*-value (moderate vs. severe and critical)
Initial laboratory finding, median (IQR)						
White blood cell/μL	6,390 (4,813–7,960)	4,080 (3,250–5,025)	3,800 (2,838–4,358)	0.006	0.043	0.669
Hemoglobin, g/dL	14.8 (14.0–15.2)	13.7 (12.8–14.9)	13.2 (12.8–14.6)	0.130	0.108	0.875
Platelets, 10^3^/μL	251.0 (216.5–287.3)	162.0 (150.5–247.5)	151.0 (118.0–201.8)	0.087	0.008	0.110
Blood urea nitrogen, mg/dL	12.0 (8.9–16.0)	14.4 (9.7–17.0)	13.5 (12.3–21.3)	0.527	0.341	0.596
Creatinine, mg/dL	0.78 (0.71–0.88)	0.69 (0.56–0.90)	0.79 (0.76–0.88)	0.363	0.730	0.281
Aspartate aminotransferase, IU/L	30.5 (20.0–36.3)	26.0 (22.0–34.0)	38.0 (29.5–77.8)	0.697	0.075	0.011
Alanine aminotransferase, IU/L	19.0 (11.8–30.3)	19.0 (11.0–31.0)	23.5 (17.3–32.3)	0.960	0.396	0.366
C-reactive protein, mg/dL	0.47 (0.13–1.23)	0.30 (0.30–1.15)	3.12 (1.25–6.63)	0.617	0.013	< 0.001
During the course of diseases, median (IQR)						
On 5–10 days from symptom onset (1 week)						
Ct value (RdRp)	35.2 (25.1–40.0)	27.9 (23.8–31.8)	26.7 (22.0–31.3)	0.044	0.034	0.588
IgG, OD_450_ ratio	0.41 (0.24–2.82)	0.42 (0.25–1.70)	0.30 (0.19–0.60)	0.861	0.113	0.143
IgM, OD_450_ ratio	0.43 (0.23–0.69)	0.41 (0.18–0.70)	0.14 (0.01–0.21)	0.734	0.219	0.254
IFN-α, pg/mL	0.0 (0.0–0.0)	0.0 (0.0–0.0)	4.1 (3.5–5.1)	0.822	0.001	< 0.001
IP-10, pg/mL	349.0 (146.2–482.8)	691.7 (333.7–1,162.0)	4,089.0 (2,291.0–6,433.0)	0.200	< 0.001	< 0.001
MIG, pg/mL	270.5 (122.7–361.5)	382.2 (282.9–584.1)	1,448.0 (663.3–2,172.0)	0.687	0.025	0.038
IL-6, pg/mL	5.1 (2.7–10.1)	11.6 (2.6–28.6)	68.3 (39.1–414.7)	0.773	0.554	0.064
IL-8, pg/mL	14.8 (8.3–20.7)	20.0 (11.9–29.8)	52.7 (32.2–292.3)	0.898	0.259	0.808
MCP-1, pg/mL	39.7 (14.9–237.3)	82.6 (48.6–173.7)	268.5 (100.2–616.7)	0.156	0.190	0.047
IFN-γ, pg/mL	4.2 (3.0–5.2)	4.1 (0.7–6.8)	7.2 (6.3–16.1)	0.987	0.095	0.006
VEGF, pg/mL	5.3 (3.1–43.8)	19.0 (7.6–28.0)	46.2 (21.1–81.6)	0.942	0.384	0.041
IL-10, pg/mL	3.6 (1.0–5.1)	3.3 (1.0–7.6)	10.0 (5.4–36.1)	0.915	0.167	0.007
On 11–16 days from symptom onset (2 weeks)						
Ct value (RdRp)	39.2 (31.2–40.0)	33.9 (29.9–40.0)	29.8 (27.2–32.8)	0.188	0.009	0.090
IgG, OD_450_ ratio	3.12 (0.88–8.12)	5.12 (1.02–9.58)	1.92 (0.41–22.07)	0.612	0.862	0.507
IgM, OD_450_ ratio	0.61 (0.53–1.10)	0.84 (0.57–1.44)	1.23 (0.43–2.02)	0.349	0.770	0.943
IFN-α, pg/mL	0.0 (0.0–0.0)	0.0 (0.0–0.0)	0.0 (0.0–0.0)	0.822	0.802	0.842
IP-10, pg/mL	223.0 (137.9–836.9)	310.9 (176.2–545.4)	2,165.0 (595.9–4,011.0)	0.208	0.296	< 0.001
MIG, pg/mL	213.7 (177.9–382.8)	382.2 (244.0–616.0)	1,270.0 (369.7–3,496.0)	0.687	0.387	0.017
IL-6, pg/mL	6.3 (1.1–24.4)	7.5 (2.2–17.8)	94.4 (31.9–565.6)	0.903	0.680	0.045
IL-8, pg/mL	8.6 (11.6–32.9)	18.8 (11.6–32.9)	83.0 (27.8–135.0)	0.494	0.330	0.005
MCP-1, pg/mL	39.1 (21.9–209.3)	47.9 (31.9–117.8)	61.8 (146.5–433.0)	0.910	0.666	0.073
IFN-γ, pg/mL	0.9 (0.0–4.1)	4.6 (2.2–8.3)	5.9 (3.2–11.7)	0.718	0.229	0.904
VEGF, pg/mL	5.3 (3.1–43.8)	19.0 (7.6–28.0)	46.2 (21.1–81.6)	0.942	0.384	0.041
IL-10, pg/mL	3.6 (1.0–5.1)	3.3 (1.0–7.6)	10.0 (5.4–36.1)	0.915	0.167	0.007
On 17–24 days from symptom onset (3 weeks)						
Ct value (RdRp)	40.0 (39.8–40.0)	38.0 (33.0–40.0)	40.0 (32.5–40.0)	0.090	0.151	1.000
IgG, OD_450_ ratio	10.02 (1.41–18.62)	15.07 (7.87–63.46)	48.27 (21.76–89.48)	0.667	0.171	0.241
IgM, OD_450_ ratio	0.93 (0.54–1.31)	1.40 (1.00–1.80)	1.92 (1.01–2.21)	0.333	0.229	0.588
IFN-α, pg/mL	0.0 (0.0–0.0)	0.0 (0.0–0.0)	0.0 (0.0–0.0)	N/A	0.842	0.704
IP-10, pg/mL	148.8 (139.0–158.6)	267.0 (164.8–375.5)	1,104.0 (335.9–2,445.0)	0.824	0.371	0.049
MIG, pg/mL	217.8 (78.3–357.3)	534.8 (295.7–836.5)	705.6 (380.2–1729.0)	0.687	0.453	0.253
IL-6, pg/mL	4.7 (0.0–9.4)	1.6 (0.0–78.3)	40.8 (7.5–47.6)	0.903	0.680	0.712
IL-8, pg/mL	40.9 (11.4–70.4)	18.2 (7.5–57.9)	43.3 (28.7–58.3)	0.898	0.706	0.458
MCP-1, pg/mL	195.0 (18.7–371.2)	34.2 (21.1–49.0)	99.3 (41.9–142.4)	0.227	0.558	0.047
IFN-γ, pg/mL	1.7 (0.0–3.5)	1.6 (0.0–17.6)	4.5 (1.6–9.6)	0.956	0.407	0.904
VEGF, pg/mL	0.0 (0.0–0.0)	19.7 (10.9–42.6)	42.4 (16.9–136.3)	0.370	0.384	0.169
IL-10, pg/mL	0.0 (0.0–0.0)	1.5 (0.1–49.0)	4.1 (1.8–7.4)	0.915	0.620	0.380
After 25 days from symptom onset (4 weeks∼)						
Ct value (RdRp)	40.0 (40.0–40.0)	40.0 (40.0–40.0)	40.0 (40.0–40.0)	1.000	1.000	1.000
IgG, OD_450_ ratio	7.83 (0.37–15.18)	11.28 (3.86–18.7)	42.77 (32.82–110.80)	0.667	0.022	0.022
IgM, OD_450_ ratio	0.32 (0.31–0.32)	0.91 (0.85–0.97)	1.79 (1.10–2.77)	0.333	0.022	0.088
IFN-α, pg/mL	0.0 (0.0–0.0)	0.0 (0.0–0.0)	0.0 (0.0–0.0)	N/A	0.842	0.842
IP-10, pg/mL	162.8 (131.1–194.4)	224.3 (208.1–240.5)	364.6 (188.7–718.9)	0.536	0.476	0.517
MIG, pg/mL	109.6 (69.4–149.9)	229.6 (204.4–394.8)	1,211.0 (448.0–3,224.0)	0.687	0.453	0.415
IL-6, pg/mL	1.2 (1.0–1.4)	3.4 (0.0–6.7)	10.4 (8.2–71.0)	0.903	0.680	0.712
IL-8, pg/mL	15.3 (4.9–25.8)	5.9 (4.1–7.7)	44.4 (24.3–90.9)	0.898	0.430	0.415
MCP-1, pg/mL	30.9 (21.8–39.9)	51.4 (48.6–54.1)	39.1 (28.6–108.5)	0.413	0.558	0.648
IFN-γ, pg/mL	3.6 (0.0–7.2)	1.9 (0.0–3.8)	3.4 (0.0–6.4)	0.977	0.971	0.904
VEGF, pg/mL	9.9 (9.5–10.3)	2.2 (0.0–4.4)	17.2 (34.5–54.4)	0.326	0.478	0.355
IL-10, pg/mL	1.3 (0.8–1.7)	1.9 (0.0–3.7)	3.5 (1.2–6.0)	0.955	0.620	0.570

Ct = threshold cycle; IL = interleukin; IFN = interferon; MCP = monocyte chemotactic protein; MIG = monokine induced by IFN-γ; N/A = not available; OD = optical density.

### Viral load kinetics.

**Figure 1.**
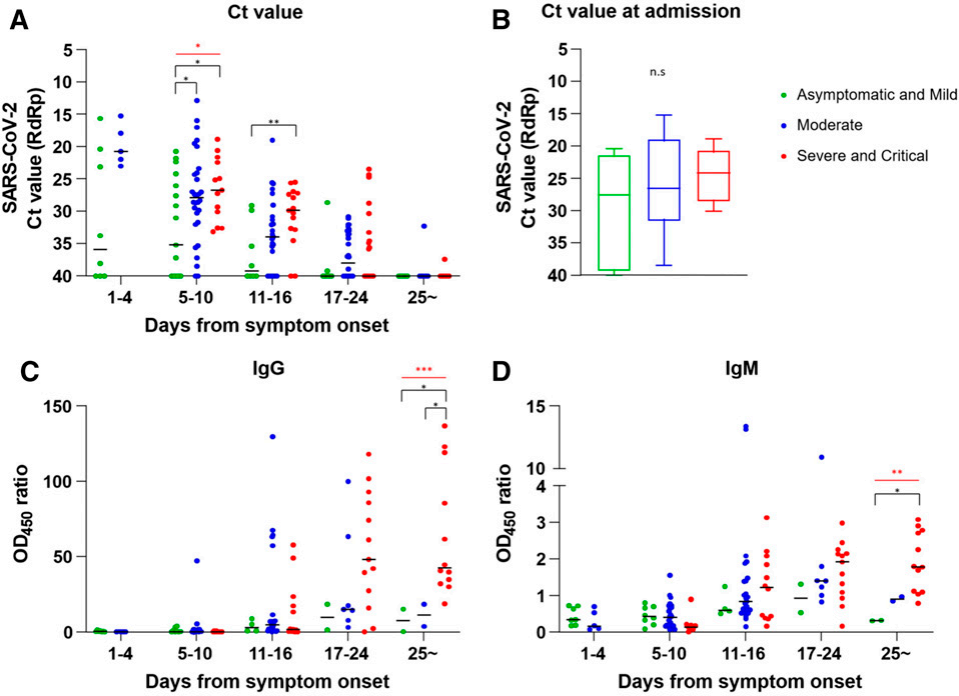
Viral load and antibody kinetics in COVID-19 patients during the course of disease. The kinetics of viral load of the upper respiratory tract from symptom onset (**A**) and the viral load at the first day of hospitalization (**B**). The kinetics of plasma IgG (**C**) and IgM (**D**) titer against SARS-CoV-2 S1 protein. If OD_450_ of 1/100 or 1/1,000 diluted sample exceeds 2.5, the endpoint titer (OD_450_ ratio) of IgG and IgM was determined by multiplication of dilution factor and OD_450_. Black significance bars compare two groups; asymptomatic and mild vs moderate, asymptomatic and mild vs severe and critical, or moderate vs severe and critical. Red bars indicate statistical differences among three groups. Blue dot = moderate; green dot = asymptomatic and mild; red dot = severe and critical. *P < 0.05, **P < 0.01, ***P < 0.001.

The detailed kinetics of viral loads in NP swab specimens are shown in Figure 1A. The viral load within 16 days from symptom onset was lowest in asymptomatic and mild group compared with those of moderate group and severe and critical group (Figure 1A and Table 2, *P* = 0.044 and 0.034, respectively). The viral load at 11–16 days from symptom onset was significantly lower in asymptomatic and mild group than that in severe and critical group (Figure 1A and Table 2, *P* = 0.009). The viral load gradually decreased over time in all patients.

The viral load on the day of admission was measured in 26 (84%) patients, and there was no significant difference in the viral load according to disease severity (Figure 1B). The initial viral load was compared at 5–10 days from symptom onset because unlike the other groups, patients in the severe and critical group were hospitalized at approximately a week later from the day of symptom onset compared with other groups. The mean (±SD) initial viral load at 5–10 days from symptom onset in asymptomatic and mild group, moderate group, and severe and critical group was 32.65 (±7.62), 27.68 (±6.98), and 26.52 (±4.82) cycles, respectively (*P* for trend = 0.038). The median time (IQR) to negative conversion of the RT-PCR result for SARS-CoV-2 from the day of symptom onset was 18 (14–24) days. The duration of positive RT-PCR results was significantly shorter in the asymptomatic and mild group than in the other groups (*P* = 0.021; Supplemental Figure 1A).

### Antibody analysis.

The plasma concentrations of SARS-CoV-2–specific IgG and IgM were measured, and the detailed kinetics of antibody titers are shown in Figure 1C and D. The median time (IQR) to seroconversion of IgG from symptom onset in the asymptomatic and mild group, moderate group, and the severe and critical group was 3 days (1.5–12.0), 11 days (7.0–13.0), and 14 days (10.0–15.8), respectively (*P* = 0.033, Supplemental Figure 1B). The plasma IgG was gradually increased and maintained during hospitalization in all groups. By contrast, the plasma IgM in the asymptomatic and mild group peaked at around 3 weeks after symptom onset, and then almost disappears and not in other groups (*P* = 0.004). Although the severe and critical group showed a relatively delayed response in terms of IgG and IgM, the response size after 25 days since symptom onset was significantly higher than those in other groups (*P* < 0.001 and *P* = 0.004, respectively).

### Cytokine analysis.

**Figure 2.**
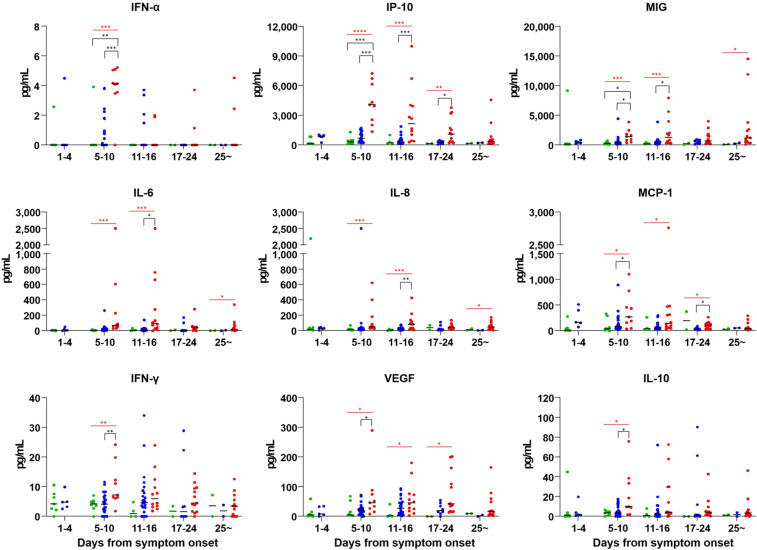
Plasma cytokines and chemokine concentrations in COVID-19 patients during the course of disease, according to the severity of illness. Black significance bars compare two groups; asymptomatic and mild vs. moderate, asymptomatic and mild vs. severe and critical, or moderate vs. severe and critical. Red bars indicate statistical differences among three groups. Blue dot = moderate; green dot = asymptomatic and mild; red dot = severe and critical. *P < 0.05, **P < 0.01, ***P < 0.001, ****P < 0.0001. IFN = interferon; IL = interleukin; IP = IFN-γ-induced; MCP = monocyte chemoattractant protein; MIG = monokine induced by IFN-γ; VEGF = vascular endothelial growth factor.

A total of 131 plasma specimens were available for multiplex cytokine bead array analysis. Among the 21 cytokines/chemokines measured, the plasma concentrations of IFN-α, IP-10, MIG, IL-6, IL-8, MCP-1, IFN-γ, VEGF, and IL-10 were found to be significantly higher in the severe and critical group than those in other groups. The detailed kinetic profiles of cytokines/chemokines in response to COVID-19 during hospitalization are shown in Figure 2 and Supplemental Figure 2. In particular, the plasma concentrations of IFN-α, IFN-γ, IP-10, MIG, and IL-6 were elevated in the severe and critical group at 5–10 days from symptom onset. Although the plasma concentrations of VEGF and IP-10 gradually decreased with time, their levels were significantly higher in the severe and critical group throughout hospitalization (Figure 2). The concentrations of other cytokines/chemokines were not significantly different according to disease severity (Supplemental Figure 2). Among 21 cytokines/chemokines, only MCP-1 had correlation with viral load (*P* = 0.034, Supplemental Figure 3). In addition, IFN-α and IP-10 had a trend to correlate with viral load (*P* for trend = 078 and 0.061, respectively).

## DISCUSSION

In our present study, we explored the kinetics of cytokine/chemokine, viral load, and antibodies in COVID-19 patients according to the disease severity. We found that viral loads in the upper respiratory tract were significantly higher in patients with severe disease than in those with mild disease between 1 week and 2 weeks from symptom onset. Moreover, compared with patients with mild disease, those with severe or critical disease had higher plasma concentrations of IFN-α, IP-10, MIG, IL-6, IL-8, MCP-1, IFN-γ, VEGF, and IL-10 between 1 week and 2 weeks from the symptom onset followed by higher antibody response after 3 weeks from symptom onset.

The pattern of viral load kinetics in COVID-19 is different from that of SARS, despite the high degree of similarity between the two viruses.^12^ Whereas the peak viral load of SARS-CoV-2 appears before symptom onset or immediately after, the highest viral load of SARS-CoV is detected after 10 days from symptom onset.^13–16^ In this study, we compared the kinetics of viral load from symptom onset according to disease severity, and found that the viral load was higher in those with more severe disease. This finding is consistent with a previous study in which respiratory viral loads were higher in patients with severe COVID-19 than those with mild COVID-19.^14^

In our study, humoral immune responses to SARS-CoV-2 developed in all patients within 2 and 3 weeks after symptom onset. The median time to seroconversion tended to be longer in the severe and critical group (approximately 2 weeks from symptom onset) than in those with milder disease. Also, the levels of IgG and IgM were significantly higher in severe COVID-19 patients, similar to the results of previous studies.^17,18^ Collectively, our results on viral load and antibody response show that the high amount of SARS-CoV-2 RNA in patients with severe disease may contribute to the induction of larger antibody response.

It is well known that excessive release of pro-inflammatory cytokines and chemokines contributes to clinical outcomes in various infections. Among the cytokine and chemokines tested in our study, the levels of IFN-α, IP-10, MIG, IL-6, IL-8, MCP-1, IFN-γ, VEGF, and IL-10 were notable in COVID-19 patients throughout the clinical course, which is consistent with the findings of previous studies.^19,20^ One study reported that the use of glucocorticoid after 7 days from symptom onset has beneficial effect on 1-month mortality, suggesting that immunopathological factors may dominate during the stage of the disease after the first week from symptom onset.^21^ Therefore, our findings that the plasma concentrations of IFN-α, IFN-γ, IP-10, MIG, and IL-6 were elevated in the severe and critical group at 5–10 days from symptom onset support that the higher plasma concentrations of pro-inflammatory cytokines after approximately 1 week from symptom onset may have a role in the enhancement of severity.

It is worthwhile to note that in our patients, the plasma concentration of IFN-α had increased around 1 week after symptom onset and then quickly dissipated. Reports on the vigor of type I IFN response in severe COVID-19 patients have shown conflicting results, with some showing impaired response^8^ and others robust response.^7^^,^^22^ However, previous studies from a limited number of COVID-19 patients could not fully reflect the cytokine responses during the varying clinical course of COVID-19. In this aspect, our current findings suggest that early increases in type I IFN response might be involved in the pathophysiology of severe COVID-19 by eliciting subsequent excessive responses of multiple cytokines and chemokines. Intriguingly, a recent longitudinal analysis showed that plasma IFN-α was sustained at high levels in patients with severe COVID-19, whereas IFN-α levels declined in those with moderate COVID-19 during their clinical course.^23^ Therefore, further studies are needed to understand the exact role of type I IFN in the pathogenesis of COVID-19.

There are several limitations to our current study. First, as a relatively small number of patients were enrolled, we could not properly investigate the factors associated with fatal outcome. Second, we did not examine SARS-CoV-2–specific T cells. It might be helpful to measure the T-cell response in COVID-19 to investigate the role of immunity in its pathogenesis and vaccine development. Third, because of mild symptoms, the date of SARS-CoV-2 infection and the date of symptom onset that patients recognized can be very different in the asymptomatic and mild group. The median time to seroconversion was too earlier in the asymptomatic and mild group; it is possible that the date of symptom onset in this group may be the middle or late stage of the disease course of COVID-19.

In conclusion, our data on viral load kinetics, antibody response, and cytokines showed that higher viral load, stronger antibody response, and excessive inflammation at 1–2 weeks from symptom onset are associated with the severity of COVID-19.

## Supplemental figures

Supplemental materials
