# Supplementary material for: Factors of Severity in Patients with COVID-19: Cytokine/Chemokine Concentrations, Viral Load, and Antibody Responses
Source: Am J Trop Med Hyg. 2020 Oct 27;103(6):2412–8. doi: 10.4269/ajtmh.20-1110 (PMC7695090; doi:10.4269/ajtmh.20-1110)

**Supplemental Figure 1. Kaplan-Meier curves for the negative conversion of viral load (A) and SARS-CoV-2-specific IgG seroconversion (B) in COVID-19 patients from symptom onset.** Seroconversion of SARS-CoV-2 IgG was analyzed in 30 patients; plasma specimens of 1 individual with mild COVID-19 were available at the 1 and 38 days from symptom onset. Green, Asymptomatic and Mild; Blue, Moderate; Red, Severe and Critical.
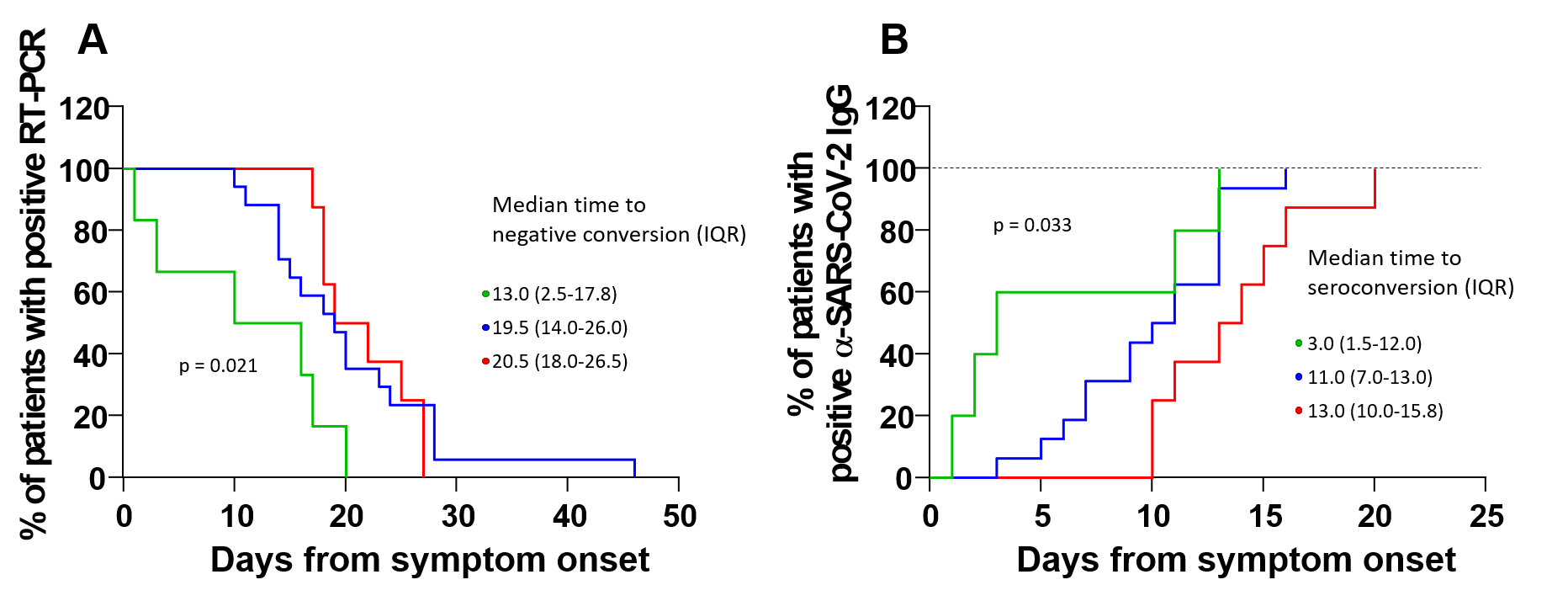


**Supplemental Figure 2. Kinetics of cytokines and chemokines over the course of the disease.** Green, Asymptomatic and Mild; Blue, Moderate; Red, Severe and Critical.
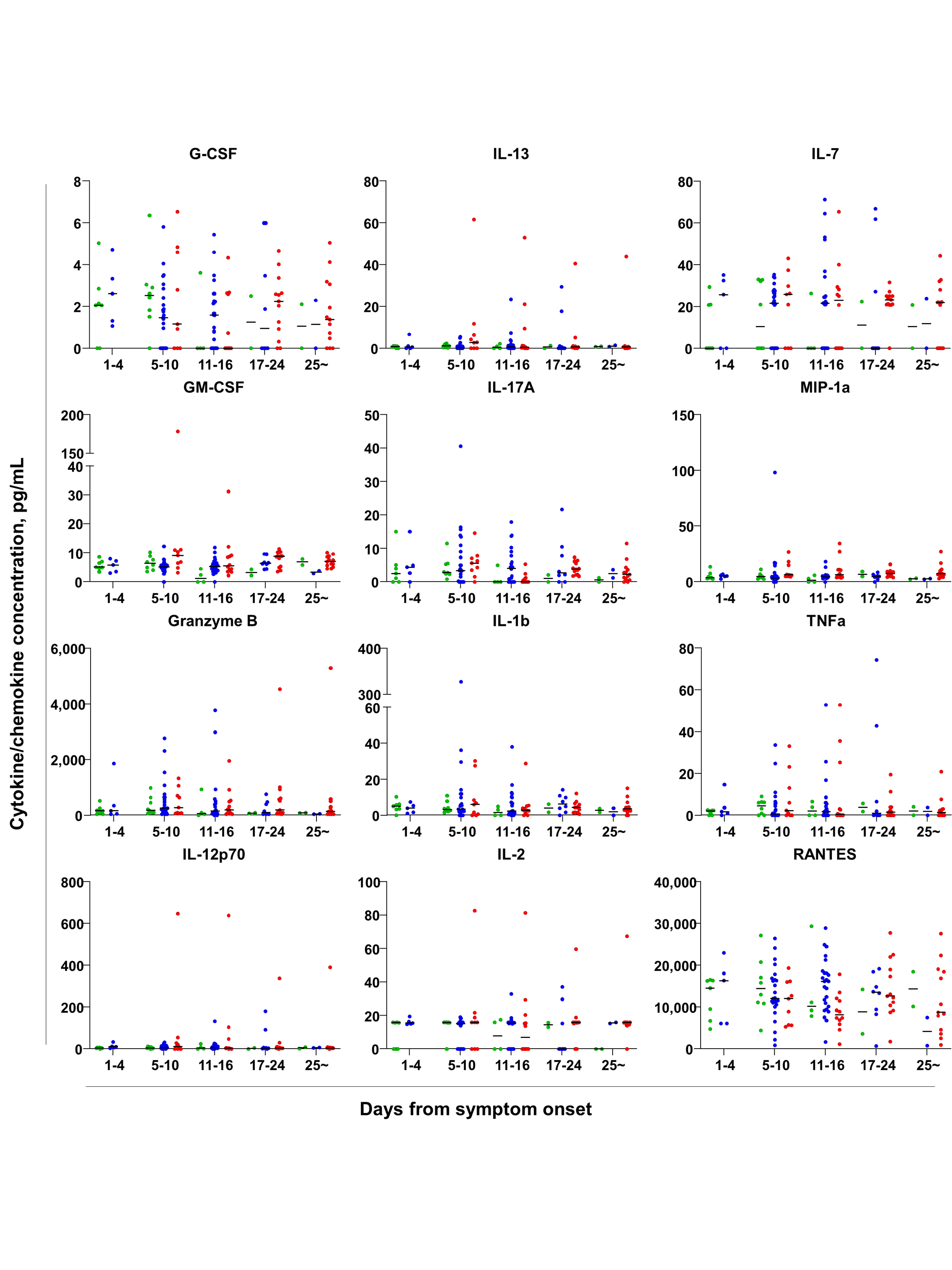


**Supplemental Figure 3. The relationship between plasma cytokine/chemokine levels and Ct value (RdRp gene) of nasopharyngeal swab specimen at the day of admission.**


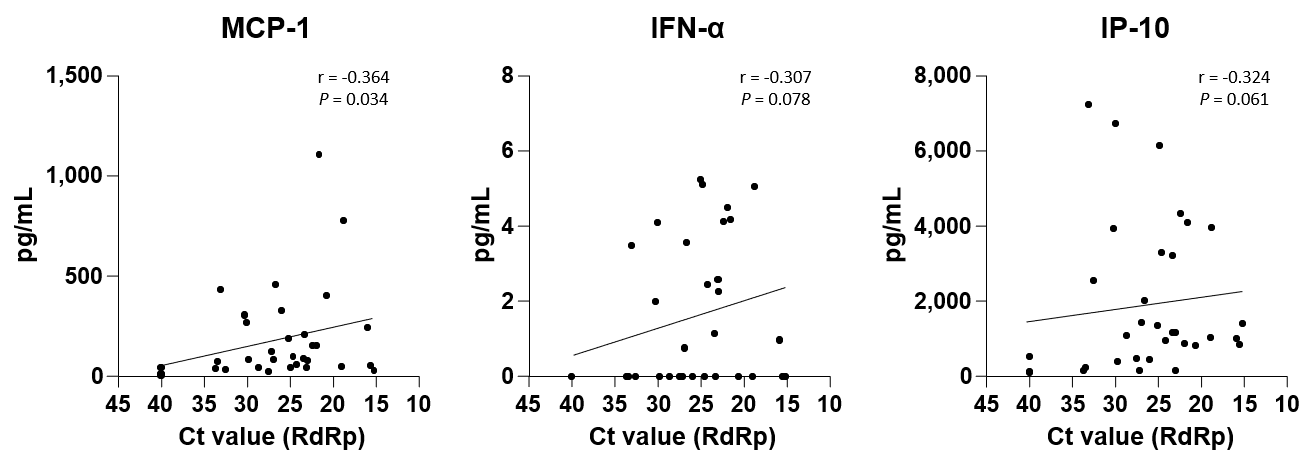

Supplement: Supplementary file 1 [file tpmd201110.SD1.doc]
